# Interferon-α-inducible Dendritic Cells Matured with OK-432 Exhibit TRAIL and Fas Ligand Pathway-mediated Killer Activity

**DOI:** 10.1038/srep42145

**Published:** 2017-02-13

**Authors:** Terutsugu Koya, Ryu Yanagisawa, Yumiko Higuchi, Kenji Sano, Shigetaka Shimodaira

**Affiliations:** 1 Centre for Advanced Cell Therapy, Shinshu University Hospital, Matsumoto, Nagano, Japan; 2 Department of Laboratory Medicine, Shinshu University Hospital, Matsumoto, Nagano, Japan; 3 Department of Regenerative Medicine, Kanazawa Medical University, Uchinada-Cho, Kahoku-Gun, Ishikawa, Japan

## Abstract

Active human dendritic cells (DCs), which efficiently induce immune responses through their functions as antigen-presenting cells, exhibit direct anti-tumour killing activity in response to some pathogens and cytokines. These antigen-presenting and tumour killing abilities may provide a breakthrough in cancer immunotherapy. However, the mechanisms underlying this killer DC activity have not been fully proven, despite the establishment of interferon-α (IFN-α)-generated killer DCs (IFN-DCs). Here mature IFN-DCs (mIFN-DCs), generated from IFN-DCs primed with OK-432 (streptococcal preparation), exhibited elevated expression of CD86 and human leukocyte antigen-DR (minimum criteria for DC vaccine clinical trials) as well as antigen-presenting abilities comparable with those of mature IL-4-DCs (mIL-4-DCs). Interestingly, the killing activity of mIFN-DCs, which correlated with the expression of CD56 (natural killer cell marker) and was activated via the tumour necrosis factor-related apoptosis-inducing ligand (TRAIL) and Fas ligand pathway, was stronger than that of IFN-DCs and remarkably stronger than that of mIL-4-DCs. Therefore, mIFN-DCs exhibit great potential as an anti-cancer vaccine that would promote both acquired immunity and direct tumour killing.

Significant advances have been made in the surgical and radiotherapeutic techniques and chemotherapeutic agents (e.g. immune checkpoint inhibitors) that comprise the field of cancer therapeutics^1^,^2^,^3^,^4^,^5^,^6^. However, the treatment of advanced cancers, which are characterized by organ involvement and distant metastasis, remains extremely difficult. Accordingly, oncological researchers have sought immunologic therapies, such as anti-tumour vaccination.

Thus far, antigen-presenting cell (APC)-based vaccination with active dendritic cells (DCs) has been evaluated as a method of efficient immunity against cancer antigens^7^. In this process, autologous monocyte-derived mature DCs are conventionally manufactured using granulocyte-macrophage colony-stimulating factor (GM-CSF) and interleukin-4 (IL-4) and are principally targeted against a specific cancer antigen^8^,^9^,^10^. However, human DC-based vaccine technology will require a breakthrough to achieve effective cancer treatment. We note that a population of cytotoxic DCs can directly kill tumour cells, in addition to inducing tumour antigen-specific cytotoxic T lymphocytes (CTLs); these ‘killer DCs’, therefore, appear to exhibit strong potential in the field of cancer immunotherapy.

DCs are generally divided into two major subtypes, myeloid DCs and plasmacytoid DCs (pDCs); however, in mice, interferon-producing killer DCs (IKDCs) have been described as a third subtype^11^,^12^. These latter cells produce substantial amounts of type I interferon (IFN) and IL-12 or IFN-γ, depending on the activating stimuli and can kill typical targets of natural killer (NK) cells via NK-activating receptors in response to stimulation with CpG^11^. In particular, B220^+^ NK1.1^+^ DCs have been reported to secrete large amounts of IFN-α and to promote the tumour necrosis factor-related apoptosis-inducing ligand (TRAIL)-dependent lysis of tumour cells^12^. In addition to IKDCs in mice, several subsets of killer DCs that exhibit anti-tumour cytotoxicity have been reported in humans^13^. These human killer DCs can be generated from monocytes *in vitro* in response to some pathogens and cytokines^14^; for example, immature IL-4-DCs (imIL-4-DCs) generated using GM-CSF and IL-4 exhibit killing activity following stimulation with bacterial lipopolysaccharide (LPS)^15^, CD40 ligand^16^ or OK-432, a streptococcal preparation^17^.

In Japan, clinical-grade OK-432 has been used as anti-tumour agent for more than 20 years, and its safety is well established^18^,^19^,^20^,^21^,^22^,^23^. Mechanistically, OK-432 promotes the functional maturation of imIL-4-DC through ligation of TLR4^24^ and TLR9^25^, and this maturation correlates with the upregulated expression of CD80, CD83 and CD86^17^,^26^,^27^, thus promoting the effective induction of antigen-specific T cells^26^. Combined treatment of mature IL-4 DCs (mIL-4-DCs) with OK-432 and prostaglandin E2 (PGE2) upregulates the expression of CD197 (CCR7), which is associated with migration to lymph nodes^27^. Katy *et al*., reported that following OK-432 activation, human mIL-4-DCs could specifically kill tumour cells via a novel CD40/CD40 ligand-mediated mechanism, without affecting normal cells^17^. OK-432 also induces the production of IL-12 from matured DCs without increasing the production of immuno-suppressive cytokines such as IL-10^17^.

Similarly, GM-CSF and IFN-α-generated DCs (IFN-DCs) also exhibit killing activity^28^,^29^. These cells express higher mRNA levels of NK cell markers, such as TRAIL, granzymes and killer cell lectin-like receptors (KLRs)^28^, and kill tumour cells via soluble TRAIL^29^, a factor that targets various tumours but does not affect normal cells^30^,^31^,^32^. According to previous reports, IFN-DCs exhibit a semi-mature phenotype, and full maturation can be achieved using CD40 ligand, LPS or polyribocytidylic acid (poly I-C)^33^,^34^. However, the effects of OK-432 on the maturation or killing activity of IFN-DCs have not previously been evaluated.

In the present study, we investigated the role of OK-432 in this setting and found that this agent not only induced the maturation, but also facilitated the killing activity of IFN-DCs. The mature IFN-DCs (mIFN-DCs) derived from OK-432-primed immature IFN-DCs exhibited similarly strong antigen-presenting abilities as mIL-4-DCs. Further study indicated that the killing activity of mIFN-DCs was stronger than that of IFN-DCs and remarkably superior to that of mIL-4-DCs. These characterizations suggest the strong clinical potential of a mIFN-DC-based vaccine for clinical use, as such an agent would be expected to induce acquired immunity and direct tumour killing.

## Results

### OK-432 induces a mature IFN-DC phenotype

**Figure 1:**
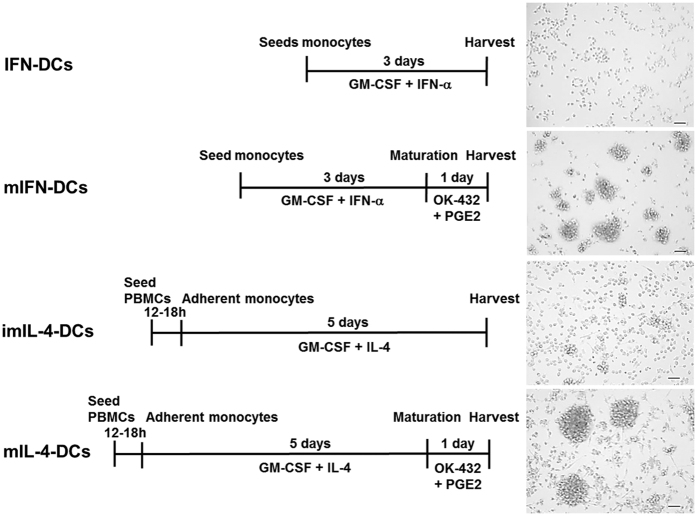
Dendritic cell (DC) preparation. IFN-DCs were generated from monocytes that were purified from PBMCs using CD14 microbeads and subjected to a 3-day non-adherent culture in the presence of GM-CSF and IFN-α. For maturation, IFN-DCs were incubated with OK-432 and PGE2 for an additional 24 h. Alternatively, imIL-4-DCs and mIL-4-DCs were prepared using a conventional adhesion protocol. Adherent monocytes were cultured with GM-CSF and IL-4 for 5 days to generate imIL-4-DCs. Subsequently, imIL-4-DCs were stimulated with OK-432 and PGE2 to yield mIL-4-DCs. During each DC preparation process, cellular morphology was observed via microscopy prior to harvesting. Black bar = 50 μm.

**Figure 2:**
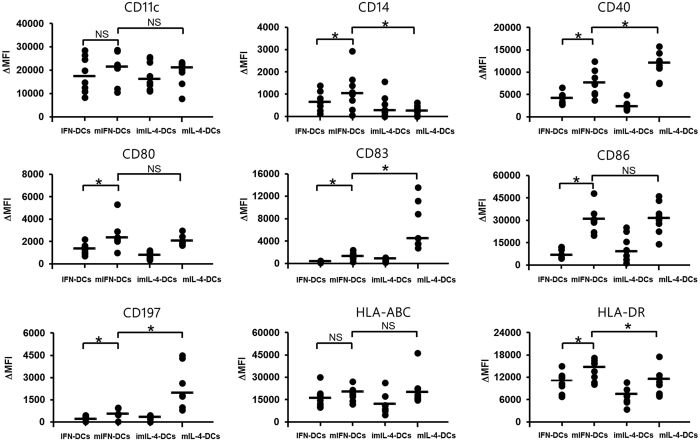
Phenotypic comparison of dendritic cells (DCs) stimulated with OK-432. DCs generated from patients (N = 8) were stained with mAbs for typical DC markers and analysed via flow cytometry. The change in mean fluorescence intensity (MFI) was calculated by subtracting the MFI values of the isotype control from the sample values. *p < 0.05; NS, not significant.

We first examined the phenotypic effects of OK-432 on mIFN-DCs and mIL-4-DCs. After OK-432 stimulation, similar remarkable clusters of cells bearing the morphologic features of mature DCs were observed equally among mIFN-DCs and mIL-4-DCs (Fig. 1). A phenotypic assessment of surface markers revealed the increased expression of costimulatory molecules such as CD80 and CD86 on mIFN-DCs, whereas the levels of these markers on mIL-4-DCs had not significantly changed (Fig. 2). Similarly, increases in the levels of CD14 and human leukocyte antigen (HLA)-DR were more evident on mIFN-DCs than on mIL-4-DCs. In contrast, mIL-4-DCs featured more strongly elevated expression of CD40, CD83 and CD197.

### Comparison of the functions of mIFN-DCs and mIL-4-DCs

**Figure 3:**
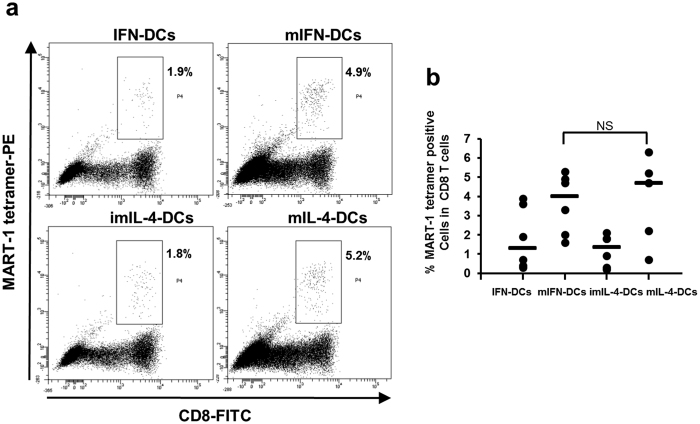
The antigen presentation ability of mIFN-DCs is similar to that of mIL-4-DCs. (**a**) Representative dot plots of data are shown. The percentages of MART-1 tetramer positive cells among CD3- and CD8-gated T cells are shown in each panel. (**b**) Summary of MART-1 CTL induction by DCs (N = 6).

DCs and MART-1 peptides were used to induce CTLs in an *in vitro* system and thus confirm the antigen presentation abilities of our DC populations. As shown in Fig. 3a, the frequency of MART-1 specific CTLs among total CD8^+^ T cells was 2.4-fold higher following mIFN-DC induction, compared to IFN-DC induction (mIFN-DCs vs. IFN-DCs = 4.9% vs. 1.9%). In contrast, the percentages of CTLs induced by mIFN-DCs and mIL-4-DCs did not differ significantly (Fig. 3b).

**Figure 4:**
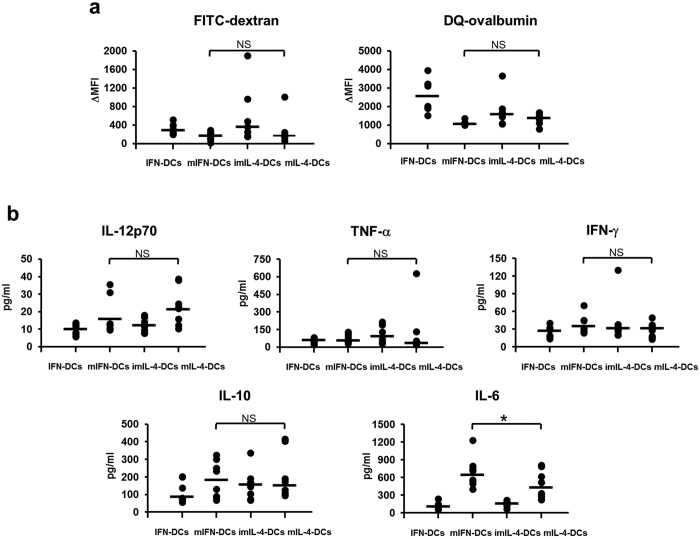
The functional analyses of DCs stimulated with OK-432. (**a**) DCs were incubated with FITC-dextran to assess pinocytotic activity (N = 6) and with DQ-ovalbumin to examine phagocytotic activity (N = 6). For each experiment, the results are expressed as the change in mean fluorescence intensity (MFI) and calculated by subtracting the MFI at 4 °C from that at 37 °C. (**b**) DCs were seeded into 24-well plates at a density of 2 × 10^5^ cells/ml for 24 h, and the supernatants were subjected to enzyme-linked immunosorbent assay (ELISA) analysis to determine the levels of IL-12p70, IFN-γ, TNF-α, IL-10 and IL-6. A summary of the cytokine production data is shown (N = 8).

FITC-dextran uptake by DCs was determined to assess pinocytotic activity. The change in mean fluorescence intensity (MFI) of FITC-dextran was slightly lower in mIFN-DCs than in IFN-DCs, similar to mIL-4-DCs (Fig. 4a, *left*). Next, DQ-ovalbumin, a self-quenched albumin that fluoresces upon proteolytic degradation, was used to demonstrate an obvious reduction in the phagocytotic activity of mIFN-DCs upon OK-432 stimulation; this was also observed in mIL-4-DCs (Fig. 4a, *right*).

Cytokine production was also investigated, using the supernatants of cells incubated in AIM-V medium for 24 h (Fig. 4b). In response to OK-432, mIFN-DCs and mIL-4-DCs produced similar amounts of IL-12p70, IFN-γ, TNF-α and IL-10. In contrast, mIFN-DCs produced significantly higher levels of IL-6, compared with mIL-4-DCs.

### mIFN-DCs possess stronger cytotoxic activity than mIL-4-DCs

**Figure 5:**
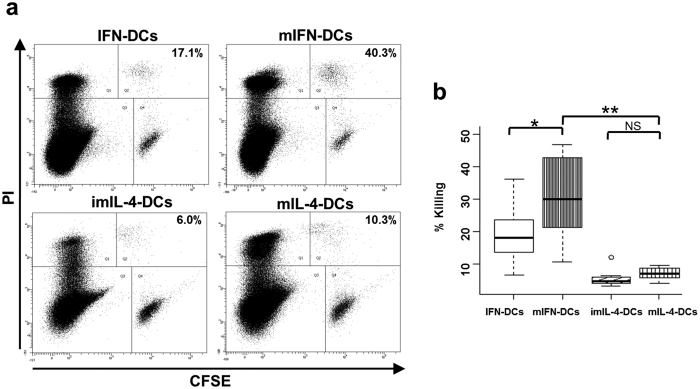
The killing activity of mIFN-DCs is stronger than that of IFN-DCs and, particularly, mIL-4-DCs. (**a**) CFSE-labelled K562 cells were incubated with indicated DCs at a ratio of 50:1 for 18 h. Incubated cells were subsequently subjected to flow cytometry and gated on a FSC and SSC dot plot to identify single cells. Subsequently, the gated cells were subgated according to CFSE and PI staining. DC killing activities are shown as percentages in the dot plot panels (representative of N = 11). (**b**) DC killing activity against K562 cells is indicated in the box plot (N = 11). *p < 0.05, **p < 0.01.

The killing activity of DCs against K562 cells was investigated using flow cytometry. In a representative experiment, IFN-DCs exhibited a killing activity of 17.1%, compared with 40.3% by mIFN-DCs (Fig. 5a), indicating a significant increase only in mIFN-DCs relative to IFN-DCs (Fig. 5b). Moreover, mIFN-DCs showed remarkably enhanced killing activity against K562 cells when compared with mIL-4-DCs. Unexpectedly, we did not observe a significant increase in tumour killing activity by mIL-4-DCs following priming with OK-432 (Fig. 5b). Accordingly, we next focused on the mechanisms underlying this enhanced tumour killing by mIFN-DCs.

### Tumour killing activity of mIFN-DCs correlates with the expression of CD56 and depends on soluble factor(s) and cell contact

**Figure 6:**
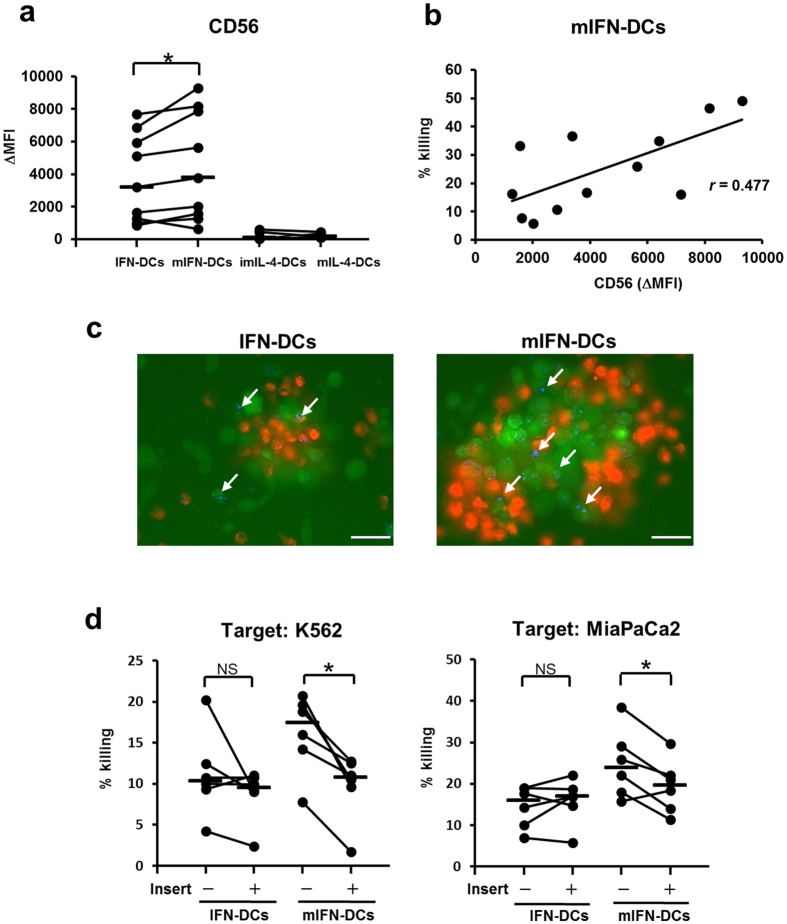
In mIFN-DCs, killing activity correlates with the expression of CD56 and is dependent on cell-to-cell contact. (**a**) The expression of CD56 on DCs is shown in terms of the mean fluorescence intensity (MFI). The expression of CD56 on mIFN-DCs and IFN-DCs was compared (N = 12). (**b**) Correlation between the expression of CD56 and mIFN-DC killing activity (N = 12). (**c**) Observed tumour killing mediated by IFN-DCs or mIFN-DCs *in vitro*. CFSE (green)-labelled K562 cells were incubated with PKH 26 (red)-stained IFN-DCs or mIFN-DCs at a ratio of 1:1. After an 18-h incubation, the cells were stained with DAPI. White arrows indicate dead cells stained with DAPI. White bar = 50 μm. (**d**) IFN-DCs or mIFN-DCs were incubated with K562 or Mia PaCa2 cells at a ratio of 50:1 and in the presence or absence of an insert membrane for 18 h (N = 6).

Relative to the expression of IFN-DCs, CD56 was significantly increased on mIFN-DCs following OK-432 treatment (Fig. 6a), and a weak correlation was observed between the expression of CD56 and the killing activity of mIFN-DCs (r = 0.477; Fig. 6b). In contrast, the expression of CD56 on imIL-4-DCs and mIL-4-DCs was extremely low, consistent with the observed weak killing activity (Fig. 6a).

Killing activity of mIFN-DCs against K562 cells was observed using fluorescence microscopy to confirm the cell contact dependency of tumour killing. IFN-DCs or mIFN-DCs were labelled with the red fluorescence dye PKH26, followed by incubation with K562 cells labelled with the green fluorescence dye CFSE. Remarkably, mIFN-DCs were found to form larger clusters with K562 cells, compared with IFN-DCs (Fig. 6c). Furthermore, large numbers of dead K562 cells (Fig. 6c: white arrows = DAPI staining) in clusters with mIFN-DCs, whereas only a few dead cells were observed in clusters with IFN-DCs. These results suggest that the formation of clusters between mIFN-DCs and target cells is important for tumour killing by mIFN-DCs. The involvement of cell contact was additionally investigated using transwell membrane assays. The killing activity of mIFN-DCs against K562 or MiaPaCa2 cells was reduced significantly in the presence of a transwell membrane insert, whereas no effect was observed for IFN-DCs (Fig. 6d). A cytotoxic assay in the presence of transwell inserts revealed that the killing activity of mIFN-DCs was reduced to a level similar to that of IFN-DCs (against K562 cells: median, IFN-DC = 9.5%, mIFN-DCs = 10.8%; against MiaPaCa2 cells: IFN-DCs = 17.0%, mIFN-DCs = 19.7%).

### mIFN-DCs exert killing activity through the TRAIL and Fas ligand pathway

**Figure 7:**
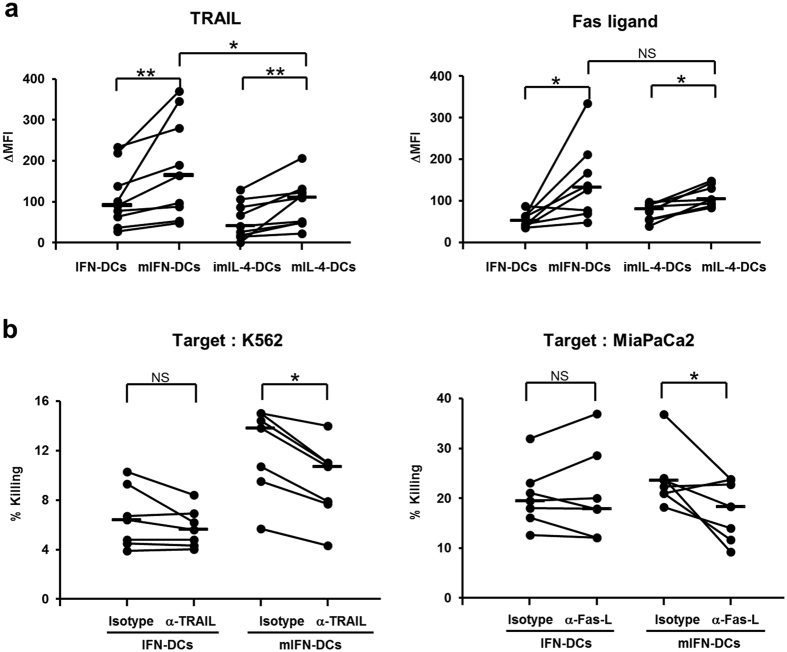
mIFN-DCs killing activity involves the TRAIL and Fas ligand pathway. (**a**) Summary of cell surface expression of TRAIL and Fas ligand (N = 9, TRAIL; N = 8, Fas ligand). (**b**) Blockade of IFN-DC or mIFN-DC killing activity with mAbs against TRAIL or Fas ligand (Fas-L). IFN-DC or mIFN-DCs were pre-treated with the indicated mAbs for 2 h, followed by incubation with CFSE-labelled K562 or MiaPaCa2 cells for 18 h at a ratio of 50:1 (N = 7).

TNF family members (TNF-α, Fas ligand, TRAIL)^35^,^36^,^37^,^38^,^39^,^40^, perforin/granzyme B^41^, NO^42^,^43^ and peroxynitrites^44^ have been reported to be involved in the tumour killing mechanisms of killer DCs. The above results suggested that the increased killing activity of mIFN-DCs in response to OK-432 depended on a cell contact mechanism, leading to the speculation of involvement by cell surface-localized cytotoxic factors in mIFN-DC-mediated tumour killing. Notably, increased cell surface expression of TRAIL and Fas ligand was observed on both mIFN-DCs and mIL-4-DCs primed with OK-432 (Fig. 7a). In particular, the expression of TRAIL was significantly higher on mIFN-DCs than on mIL-4-DCs, whereas both cell populations exhibited similar increases in the expression of Fas ligand.

We subsequently examined whether the tumour killing activity of mIFN-DCs was dependent on TRAIL and Fas ligand. K562 (TRAIL sensitive)^17^ and MiaPaCa2 (Fas sensitive) cell lines^45^ were treated with antibodies specific for TRAIL or Fas ligand, respectively, to test the blocking of each ligand. TRAIL antibody blockade significantly suppressed the killing of K562 cells by mIFN-DCs (Fig. 7b, *left panel*), whereas this treatment had no significant effect on the killing of K562 cells by IFN-DCs. Similarly, Fas ligand antibody blockade reduced the killing of MiaPaCa2 cells by mIFN-DCs (Fig. 7b, *right panel*), whereas this treatment did not influence IFN-DCs-mediated killing. These results suggested that, in contrast to IFN-DCs, the killing activity of mIFN-DCs is mediated through the TRAIL and Fas ligand pathway.

## Discussion

OK-432 has been well established as a clinical agent for DC maturation^18^,^19^,^20^,^21^,^22^,^23^. Accordingly, a 4-day non-adherent culture system in which IFN-DCs were primed with OK-432 was developed to generate mIFN-DCs. In contrast to mIL-4-DCs, the short-term generation of mIFN-DCs is also clinically beneficial because although mIFN-DCs express lower levels of CD40 and CD83, the increased expression levels of CD86 and HLA-DR, which were similar to those of mIL-4-DCs, met the minimum release criteria for DC vaccines^46^. Based on the pinocytotic and phagocytotic activity, cytokine production and antigen presenting profiles of mIFN-DCs were found to correlate with those of mature DCs, similar to mIL-4-DCs. Interestingly, OK-432 also facilitated an increase in the killing activity of IFN-DCs. However, the killing activity of mIFN-DCs, which correlated with the expression of CD56, was stronger than that of both IFN-DCs and mIL-4-DCs. OK-432 upregulated the expression of CD56 and cell surface TRAIL and Fas ligand on mIFN-DCs, and the independent involvement of TRAIL and Fas ligand in mIFN-DC-mediated tumour killing was proven via specific antibody blockade.

Although OK-432-activated human DCs kill tumour cells through CD40/CD40 ligand interactions^17^, other factors that may be involved in this cytotoxic process have not been fully examined. The association of CD56 with cell adhesion^47^,^48^ suggested that tumour killing by mIFN-DCs with expression of CD56 would depend on cell contact. We further speculated that cell surface cytotoxic molecules, such as tumour necrosis factor (TNF) family members, might participate in the required cell-to-cell interactions. Although OK-432 stimulation was previously found to upregulate the expression of Fas ligand on mononuclear cells^49^, our report is the first to demonstrate the increased expression of CD56, cell surface TRAIL and Fas ligand on mIFN-DCs following OK-432 stimulation. The observed clustering of mIFN-DCs and tumour cells might facilitate tumour killing via either cell surface TRAIL or Fas ligand. The reduced killing activity of mIFN-DCs in the presence of anti-TRAIL antibody suggests that these cells use mechanisms other than CD40/CD40 ligand signalling to exert their cytotoxic effects^50^. In contrast to previous reports, however, OK-432 did not promote a significant increase in tumour killing by mIL-4-DCs, possibly because of differences in the maturation cocktail used in combination with OK-432 and PGE2, as well as the incubation time^17^; both factors might have affected tumour killing by OK-432-induced DCs.

Clearly, OK-432-induced mIFN-DCs exhibited remarkably strong killing activity, which correlated with the expression of CD56 and depended on TRAIL and Fas ligand. Previously, CD56^+^ IFN-DCs exhibited stronger cytotoxicity against K562 cells, the majority of which are TRAIL positive, when compared with CD56^−^ IFN-DCs^29^. However, in our study, we did not observe significant suppression of tumour killing by IFN-DCs in response to anti-TRAIL antibody treatment. The observed expression of granzyme B^28^ suggests that IFN-DCs might use multiple soluble factors to kill tumour cells. However, IFN-DCs are known to share phenotypic features with pDCs (e.g. CD11c and CD123)^29^, and pDC tumour-killing also correlates with the expression of TRAIL and CD56. Furthermore, the expression of TRAIL and TRAIL-dependent cytotoxicity are upregulated in pDCs in response to HIV infection^51^. pDCs activated by the tick**-**borne encephalitis vaccine FSME specifically upregulate the expression of CD56, which coincides with elevated levels of cytotoxic molecules such as TRAIL; furthermore FSME-activated pDCs specifically lyse NK target cell lines in a cell-cell contact dependent manner^52^. Accordingly, OK-432-induced mIFN-DCs might exhibit pDC-like responses.

It remains to be determined whether mIFN-DCs can kill tumour cells and induce CTLs *in vivo*. As mentioned above, mIFN-DCs express CD86 and HLA-DR^46^ and thus potentially possess antigen presentation ability comparable to that of mIL-4-DCs. However, the low level of expression of CD197 on mIFN-DCs might not be sufficient to promote migration to the lymph nodes and subsequent CTL induction. In addition to the ability to migrate to specific regions for direct tumour killing, the ability of antigen-pulsed mIFN-DCs to induce CTLs should be examined in tumour-bearing animal models. A report by Padovan *et al*., in which IFN-DCs produced IP10/CXCL10 and MIG/CXCL9 to attract activated CD8^+^ T cells and thus expand CTLs efficiently^53^, led to speculation that mIFN-DCs could potentially induce CTLs directly in the tumour regions, rather than in the lymph nodes. We further note that we observed higher levels of IL-6 in cultures of mIFN-DCs, compared with mIL-4-DCs (Fig. 4b). IL-6 mediates febrile and acute-phase responses by upregulating chemokines and cell adhesion molecules that support the growth of B cells and Th17 cells, but suppress regulatory T cells (T regs)^54^. Accordingly, the observed high production of IL-6 might provide mIFN-DCs with an advantage with respect to the induction of CTLs in tumour regions, which tend to harbour T regs^55^.

Clinically, mIFN-DCs may directly kill tumour cells that retain sensitivity to TRAIL and Fas ligand signalling. Unlike normal cells, various types of tumours are susceptible to TRAIL-induced apoptosis, and the Fas/Fas ligand-mediated killing activity of mIFN-DCs might be less advantageous for cancer treatment. Fas/Fas ligand interactions are known to induce apoptosis in hepatocytes^56^ and to have immunomodulatory effects on immune homeostasis and tolerance^57^. Although TRAIL- or Fas ligand-mediated apoptosis-resistant cancer cells have been reported, one option for cancer therapy would combine the anti-tumour effects of TRAIL and Fas ligand with standard chemotherapy^58^,^59^.

**In conclusion, this study** confirms **that OK-432 induced** the **maturation** of **IFN-DCs with** strong **antigen-presenting** abilities (**comparable to mIL-4-DCs**), and facilitated the **TRAIL- and Fas ligand**-mediated killing activity of these mIFN-DCs. Further clarification of **the characteristics of mIFN-DCs** is expected to **contribute to the development** of adaptive immunotherapies and the **achievement of acquired** anti-cancer **immunity**.

## Methods

### Subjects and Ethics Statement

DC vaccination therapy at the Shinshu University Hospital was approved by the Ethics Committee of Shinshu University School of Medicine (approval number 1123 and 1199). The Act on the Safety of Regenerative Medicine in Japan was enforced since November 25, 2014. Class III technologies use somatic cells with accumulated clinical experiences and are regarded as low risk technologies. The DC vaccination therapy (Class III technology) at the Shinshu University was approved on November 25, 2015 (approval number: PC3150643 and PC3150645). Human peripheral blood mononuclear cell (PBMC)-rich fraction was collected from blood samples of patients via leukapheresis with a COM.TEC cell separator (Fresenius Kabi Japan K.K., Tokyo, Japan). PBMCs were subsequently isolated using a Ficoll-Plaque Premium (GE Healthcare, Piscataway, NJ, USA) density gradient. The collection and use of blood complied with relevant guidelines and institutional practices from Ethics Committees of Shinshu University School of Medicine (approval number 2107). Written informed consent was obtained from all patients. All methods in this study were performed in accordance with the Ethical Guidelines for Medical and Health Research involving Human Subjects proposed by Ministry of Health, Labour and Welfare in Japan^60^.

### DC generation

To generate IFN-DCs, monocytes were purified from PBMCs using CD14 microbeads (Miltenyi Biotec, Bergisch Gladbach, Germany), which yielded a CD14^+^ monocyte purity of >95%. The monocytes were cultured in AIM-V medium (Gibco, Gaithersburg, MD, USA) containing GM-CSF (1,000 U/ml; Miltenyi Biotec) and PEGylated-IFN-α-2b (1 μg/ml of PEG-Intron; MSD, Tokyo, Japan) for 3 days in a HydroCell (Cell Seed, Tokyo, Japan).

For maturation, IFN-DCs were stimulated with OK-432 (10 μg/ml; Chugai Pharmaceutical Co, Ltd, Tokyo, Japan) and PGE2 (10 ng/ml; Daiichi Fine Chemical Co, Ltd, Toyama, Japan). After a 24-h incubation, mIFN-DCs were harvested for further analysis. Alternatively, imIL-4-DCs were generated using a previously reported adhesion protocol^8^,^9^,^10^. In brief, PBMCs were placed into 100-mm plastic tissue culture plates (BD Biosciences, San Jose, CA, USA) in AIM-V medium. After removing non-adherent cells, 50 ng/ml of GM-CSF (Gentaur, Brussels, Belgium) and 50 ng/ml of IL-4 (R&D Systems, Inc., Minneapolis, MN, USA) were added the following day, and the cells were cultured for 5 days to generate imDCs. ImIL-4-DCs were subsequently stimulated with 10 μg/ml of OK-432 and 50 ng/ml of PGE2 for 24 h to generate mIL-4-DCs.

### Phenotyping of DCs

Fluorescein isothiocyanate (FITC)- or phycoerythrin (PE)-conjugated forms of monoclonal antibodies (mAbs) against the following DC markers were used: CD11c, CD80, CD86 and HLA-ABC (BD Pharmingen, San Diego, CA, USA); CD14, CD40, CD83 and HLA-DR (eBioscience, San Diego, CA, USA) and CD197 (R&D Systems, Minneapolis, MN, USA). To detect NK cell markers, DCs were incubated with allophycocyanin (APC)-conjugated anti-CD56 mAb (BD Pharmingen, San Diego, CA, USA). To evaluate cytotoxic cell surface molecules, PE-conjugated mAbs specific for anti-TRAIL (BD Pharmingen) and anti-Fas ligand (eBioscience) were used. All analyses were performed on a FACSCanto II flow cytometer (BD Biosciences).

### CTL induction *in vitro*

PBMCs from HLA-A^*^02:01 patients were used to generate DCs, which were subsequently pulsed with 20 μg/ml of MART-1 _26-35_ A27L (ELAGIGILTV) peptide (MBL, Medical & Biological Laboratories Co., Ltd., Nagoya, Japan) at 37 °С for 1 h. After washing with AIM-V medium, DCs were treated with mitomycin C (25 μg/ml; Kyowa Hakko Kogyo Co, Ltd, Tokyo, Japan) at 37 °C for 1 h. After two washes, the prepared DCs were used as stimulator cells; non-adherent PBMCs collected after seeding into plastic tissue-culture plates were used as responder cells. Stimulator (1 × 10^6^) and responder cells were co-cultured at a ratio of 1:10 in AIM-V medium supplemented with IL-2 (2.5 U/ml; Imunace, Shionogi, Pharmaceutical, Osaka, Japan), IL-7 (5 ng/ml; R&D Systems) and IL-15 (10 ng/ml; PeproTech, Rocky Hill, NJ, USA) for 3−5 days. AIM-V media supplemented with 10% foetal bovine serum (FBS; Thermo Fisher Scientific K.K., Yokohama, Japan) was added depending on cell expansion. After a 2–3-day incubation, cells were harvested and stained with FITC-conjugated anti-CD8 (Beckman Coulter, Inc., Brea, CA, USA) and APC-conjugated anti-CD3 (eBioscience) mAbs and T-select HLA-A*02:01 MART-1 tetramer-ELAGIGILTV-PE (MBL).

### Pinocytotic and phagocytotic assay

To evaluate pinocytotic activity, DCs were incubated with 250 μg/ml FITC-dextran (Molecular Probes, Eugene OR, USA) in AIM-V medium supplemented with 10% FBS at 37 °С for 3 h and subsequently washed twice with FACS buffer. DCs were then incubated with 10 μg/ml DQ-ovalbumin (Molecular Probes, Eugene OR, USA) at 37 °C for 30 min to examine phagocytotic activity. After two washes, cells were analysed using flow cytometry.

### Cytokine production

DCs were seeded at a density of 2 × 10^5^ cells/ml into a 24-well plate. After a 24-h incubation in AIM-V medium, cytokines were measured in the collected supernatants using ELISA kits for IL-12p70, IFN-γ (R&D Systems), TNF-α, IL-6 and IL-10 (BD Biosciences) according to the manufacturers’ protocols.

### Cytotoxicity assay

To examine the cytotoxic activity of DCs, we used a previously reported method^28^ with slight modifications. Initially, 1 × 10^6^ K562 (ATCC, Manassas, VA, USA) or MiaPaCa2 cells (RIKEN BRC, Tsukuba, Japan) were labelled with carboxyfluorescein succinimidyl ester (CFSE; 5 μM; Molecular Probes) in PBS supplemented with 0.1% FBS at 37 °C for 10 min, followed by two washes with AIM-V medium. Different numbers of DCs were used as effectors and were incubated with 1 × 10^4^ CFSE-labelled K562 or MiaPaCa2 cells (targets) in AIM-V medium containing 10% FBS at 37 °C for 18 h. To evaluate intrinsic controls, an aliquot of K562 or MiaPaCa2 cells equivalent to the number of effector cells was incubated with labelled target cells. The cells were then washed in FACS buffer, stained with propidium iodide (PI; 2 μg/ml; Sigma-Aldrich Co. LLC., Tokyo, Japan) for 5 min and analysed using flow cytometry. Tumour-specific killing activity was determined as follows: % killing = experimental % of PI^+^ CFSE-labelled cells − intrinsic % of PI^+^ CFSE-labelled cells. To assess contact-dependent killing, CFSE-labelled target cells and effector cells were separated using transwell inserts fitted with 0.4-μm pore polyester membranes (Corning Inc., Corning, NY, USA). For the blocking experiment, effector cells were pre-treated with 10 μg/ml anti-TRAIL or anti-Fas ligand mAbs (Biolegend, San Diego, CA, USA) at 37 °C for 2 h and were incubated with labelled target cells.

### Fluorescence microscopy

IFN-DCs or mIFN-DCs were labelled with the red fluorescence dye PKH26 (2 μM; Sigma-Aldrich) according to the manufacturer’s instructions, and subsequently incubated with CFSE-labelled K562 cells at a ratio of 1:1. After an 18-h incubation, cells were stained with DAPI (1.5 μM; Molecular Probes) for 5 min to detect cell death and assessed under a fluorescence microscope.

### Statistical analysis

The Wilcoxon signed-rank test with Bonferroni correction was used to compare differences among groups. Spearman’s rank-order correlation was used to calculate the correlation of the expression of CD56 with killing activity. All statistical analyses were performed using IBM SPSS Advanced Statistics software, version 23.0 (IBM Japan, Tokyo, Japan). Differences were considered statistically significant at a *p* value < 0.05.

## Additional Information

**How to cite this article**: Koya, T. *et al*. Interferon-α-inducible Dendritic Cells Matured with OK-432 Exhibit TRAIL and Fas Ligand Pathway-mediated Killer Activity. *Sci. Rep.* **7**, 42145; doi: 10.1038/srep42145 (2017).

**Publisher's note:** Springer Nature remains neutral with regard to jurisdictional claims in published maps and institutional affiliations.
